# Book Review

**Published:** 1895-09

**Authors:** 


*Wintering in Egypt.* By ARTHUR J. M. BENTLEY, M.D. and Rev. C. G. GRIFFINHOOFE, M.A. Pp. 188. London: SIMPKIN, MARSHALL, HAMILTON, KENT & Co., LTD. 1894.

This eminently practical and most graphic sketch of life in Lower Egypt will prove of great service to physicians who contemplate sending patients to Egypt, as well as to all who are about to start for that interesting land. It contains much information of a useful character on the alternative routes, their cost, and the kind of outfit that travellers should provide. Dr. Bentley's remarks on the climate of Egypt and the class of cases that will be benefited by a stay there are clearly and concisely expressed, and exhibit a judicious moderation and cautious judgment.

Dr. Bentley is careful to give us his summer and winter addresses, but the authors have not taken the trouble to provide their book with an index.

*Anatomy and Art.* By ROBERT FLETCHER, M.D. Pp. 24. Washington: JUDD & DETWEILER. 1895.—The question whether Art has received any considerable aid from Anatomy is discussed by Dr. Fletcher in a light and interesting, but rather discursive, manner in this address, which he read before the Philosophical Society of Washington on December 12th, 1894. He comes to the conclusion that Egyptian and Greek sculptures and bas-relief owed nothing to knowledge of the structure of the body, and that the most perfect works of Pheidias and his contemporaries were entirely copies from models often idealised, but quite independent of anatomical knowledge deeper than surface markings. It was not until the Italian renaissance that dissection of muscles, veins, &c., began to be used by artists, and the result was seen in minute attention to anatomical detail. Whether they really gained much by this is an open question, which is only touched upon in this pamphlet.

*Diphtheria and its Treatment.* By BROWNLOW R. MARTIN, M.B. Second Edition. Pp. 64. London: BAILLIÈRE, TINDALL AND COX. 1895.—The author has decidedly improved on the first edition by cutting out the advertisements, which formerly exceeded the text in number of pages, and by giving statistics of cases treated by his method, which consists in administering sulphite of magnesium internally and insufflating it locally. The treatment has been successful in other hands as well as his own, and seems to be deserving of further trial.

*Laboratory Guide for the Bacteriologist.* By LANGDON FROTHINGHAM. Pp. 61. Philadelphia: W. B. SAUNDERS. 1895.—It is scarcely possible to practise the various operations necessary for the examination of micro-organisms without experiencing an almost irresistible desire to vary the prescribed procedure in innumerable ways; and although few of these experimental variations may ultimately prove to be advantageous, it is very desirable to keep brief notes of what has been done. It is further very convenient to have standard methods collected in a compact form for easy reference in the laboratory. These requirements are satisfactorily met by the work under notice, consisting as it does of clearly-printed concise descriptions of the various processes, interleaved with blank pages for annotations. Absolute accuracy of detail is above all things necessary here, and a fairly close inspection has resulted in the discovery of one important error only—the proportion of potash in Löffler's methylene blue solution being increased tenfold. The only portion of this book laying claim to originality is the introduction, which consists of suggestions as to the most convenient and rapid way of operating: these notes are thoroughly practical, and, with the exception of a curious confusion between the terms "slip" and "cover-glass," admirably clear. There is perhaps a tendency to sacrifice too much to secure the utmost rapidity of execution: for instance, with such material as tubercular sputum, spreading on the cover-glass with a needle will not satisfactorily replace squeezing out between two covers.

*Urinary Surgery.* By E. HURRY FENWICK. Pp. 219. Bristol: JOHN WRIGHT & Co. 1894.—The surgeon is always anxious to know the advance which modern surgery is constantly making. In the department of urinary surgery this need is well supplied by this work of Mr. Fenwick's. Those who have time can, by means of his numerous references, turn to the original articles from which the book is a compilation; but to the busy surgeon the information given in the book as to diagnosis and new methods of treatment will be of great value. We often want a book to supplement the last editions of the leading text-books, and this Mr. Fenwick's does in his special department. The author contributes an original article on tumours of the bladder, and electric urethroscopy, and the use of the cystoscope is fully described. We welcome the book as one of great practical value. It is the first of a series of "Epitomes of Modern Surgical Progress."

*Syllabus of Gynecology, based on the American Text-Book of Gynecology.* By J. W. LONG, M.D. Pp. 133. Philadelphia: W. B. SAUNDERS. 1895.—If we must have a syllabus of gynecology, no doubt it is well to choose the American text-book as the standard; but we are amongst the number of those who think that works of this kind are better made by students and practitioners themselves. When prepared for us by others, their use is very limited, and many students are apt, in the perusal of such condensed pages as these, to snatch at the shadow, and lose the substance of the larger manuals. Apart from these opinions, the work has been carefully compiled and planned in a most clear manner, and leaves little to be desired. The book has been interleaved, which will render it more useful for the purpose of note-taking. Typographical errors seem scarce, but we saw "Bacillus colis commune" on page 7.

*The Mother's Help and Guide to the Domestic Management of her Children.* By P. MURRAY BRAIDWOOD, M.D. Pp. viii., 134. London: THE SCIENTIFIC PRESS, LIMITED. 1894.—Dr. Braidwood appears to have carefully avoided the objectionable features that are often found in books of this kind; but whether this will prove advantageous to the sale of his book is another matter. The rules laid down for the mother's guidance are clearly and concisely stated, and amongst the suggestions are very few to which we can take exception. We can confidently recommend the book for the purpose for which it is intended, and we think it contains many hints that would be valuable to the junior practitioner. The style of its publication leaves nothing to be desired.

*Essentials of Practice of Pharmacy.* By LUCIUS E. SAYRE, Ph.G. Second Edition. Pp. ix., 17-200. Philadelphia: W. B. SAUNDERS. 1894.—This work is intended for pharmaceutical, rather than medical, students; and it is based on the United States Pharmacopœia. It is in the form of questions and answers, which are well-arranged, and the information is concise and clearly put; but there is evidence of carelessness in the correction of proof-sheets, especially in regard to chemical formulæ and reactions.

*Notes on the Newer Remedies.* By DAVID CERNA, M.D., Ph.D. Second Edition. Pp. 253. Philadelphia: W. B. SAUNDERS. 1895.—It must be exceedingly difficult to keep pace with the very large number of new drugs mentioned in this work. The author has given an excellent epitome of what is known, but no one person can be expected to use so many weapons and to use them efficiently. Those who are in search of new drugs will find all they can wish for here, and will have a reliable guide for investigation in little-known fields.

*The Droitwich Brine Baths as Therapeutic Agents in Various Diseases.* By W. H. TOMLINS. Pp. 22. London: H. K. LEWIS. 1895.—The author of this pamphlet endeavours to show that Droitwich may justly claim a much higher place than it has as yet secured as a health-resort.

*Food for the Sick and Convalescent.* By MARY FEACHEM. Pp. 8. Bristol: ROSE AND HARRIS. 1895.—This booklet contains a number of recipes for the making of ordinary foods for the invalid. The instructions are simple and easy to follow: the results should be excellent. The authoress is a Diplomée First Class, South Kensington, and her instructions appear to be founded on the elementary principles of chemistry, which in relation to cooking are frequently either forgotten or ignored.

*The Prevention of Small-pox, with Special Reference to the Origin and Development of the Stamping-out System.* By EDGAR M. CROOKSHANK, M.B. Pp. 40. London: H. K. LEWIS. 1894.—We have very little to say about this pamphlet, which consists of an inaugural address delivered before the Medical Society of King's College. The history of the Plague in London is well and tersely written, and interested us much; and we should heartily rejoice at the foundation of an "International Board of Health." This, if properly conducted, should be productive of nothing but good; but this would not include giving up vaccination. We contend that vaccination and re-vaccination should go hand-in-hand—or better, arm-to-arm—with all systems of isolation and notification. When the whole of Europe is determined to isolate and thus limit the spread of small-pox infection, then the time might come when vaccination would be of less import; but that time is far distant yet.

*The Sick Poor in Workhouses.* First Series. Pp. xiii., 53. London: SMITH, ELDER & Co. 1895. This pamphlet is composed of twelve reports on the state of our provincial workhouses and infirmaries, by a special commission of the *British Medical Journal*, with an introduction by Mr. Ernest Hart. As they have already been published in the *Journal* of the Association, the main facts are known to most of our readers. Mr. Hart's excellent work in conjunction with the late Drs. Anstie and Joseph Rogers on the London Infirmaries in 1865 is still fresh in the minds of many, but it is only too clear that a new crusade is urgently needed. After reading the accounts of the infirmaries in 1865, one is surprised to find, thirty years after, almost the same conditions prevailing. That such a state of things existed, or could exist, is a revelation to many, and the British Medical Association is to be heartily congratulated on the exposure it has made. We are glad to observe that many Boards of Guardians have taken the criticism to heart, and are thinking, if nothing more, of putting their houses in order. It is evidently time they did. The commission gives praise when it is due.

*The Johns Hopkins Hospital Reports.* Vol. IV., Nos. 4–5. *Report in Neurology*, II. Baltimore: The Johns Hopkins Press. 1894.—This volume consists of papers by Dr. Berkley detailing the results of researches, made by Golgi's chrome-silver method, on the finer nerve-supply of various organs. It would be impossible to give in detail the important results arrived at for each organ; but those relating to the lung may be briefly mentioned in order to show how these investigations bear out, and offer an anatomical basis for, deductions long since made from physiological and clinical researches. The main nerve-supply of the lung is derived from nerve plexuses which follow the bronchial arteries; from these plexuses fine nerve-fibres pass off to end in the coats of the bronchial tubes, as small rounded end-knobs in the closest apposition to the plain muscular fibres, and as fine terminations in the mucous membrane, some ending in the subepithelial tissues—others, more particularly in the smaller bronchi, in the cement spaces between the epithelial cells. The pavement epithelium of the pulmonary alveoli has no nerve supply. This evidently harmonises with received opinions that interchange of gases between the alveoli and the blood capillaries is in the main, if not entirely, a mechanical one, dependent on the relative tensions of oxygen and carbon dioxide in the air and blood. It also supports deductions made from the study of asthma that the bronchial musculature has an extensive motor nerve-supply, and that the bronchial mucous membrane is extremely sensitive to the quality of the inspired air. Equally important results for the cardiac ventricles, the thyroid, the pituitary gland and the submaxillary gland are also given by Dr. Berkley, whose papers merit most careful study.

*The Middlesex Hospital. Reports of the Medical and Surgical Registrars, and Pathologist for the year* 1893. London: H. K. Lewis. 1894.—These reports contain the usual account of the work done at the Middlesex Hospital in the various departments during the year 1893, with a detailed account of the more interesting and important cases. It thus forms a valuable store-house of clinical observation. In addition there are several important abstracts by the Surgical Registrar. We especially notice those on cases of strangulated hernia and perityphlitis during the years 1890–1893 inclusive; another on fourteen cases of laparotomy for exploration and other purposes; and a series of abstracts of operations on the kidney for fourteen years from the original operation of nephrolithotomy in 1880 to the end of 1893, which comprises the details of 118 operations, and cannot fail to be of great value to all interested in renal surgery.

*The Westminster Hospital Reports.* Vol. IX. London: J. & A. Churchill. 1895.—This volume reminds us that during the past year two well-known Westminster men—Octavius Sturges and Barnard Holt—have died, and the book contains a fitting eulogy of each, with an excellent portrait of Dr. Sturges. The ground covered by these reports is a wide one, and there is much interesting material brought together from the various branches of the profession. Speaking generally, the articles and reports are well written. Under the able editorship of Dr. R. G. Hebb, this volume fully maintains the reputation of the series.

*Transactions of the Medical Society of London.* Vol. XVII. London: Harrison and Sons. 1894. This volume contains papers of exceptional interest on the association between variations of pulse, Graves's disease, epileptiform convulsions, and infantile convulsions. Dr. Bristowe relates a series of cases in which fits of an epileptiform character were induced by extreme slowness of pulse. Mr. Arthur Maude's papers on Graves's disease all tend to the conclusion that "the universal goitre, the connection with myxœdema, and the results of operations on the thyroid, point to that gland as the fountain head of evil." Dr. Alexander Morison points out that the cardiac state may be regarded as an important key to the treatment of infantile convulsions. Mr. Frederick Treves's Lettsomian lectures on Peritonitis are reprinted in this volume, and the starting points of tuberculous disease in children are described by Dr. J. Walter Carr. The fact that perforation of a chronic ulcer of the duodenum has been successfully treated by excision in the case related by Mr. Henry Percy Dean is worthy of being recorded, and gives encouragement to surgical treatment of the not uncommon cases of perforation from gastric ulcer or from typhoid fever.

*Medico-Chirurgical Transactions.* Vol. LXXVII. London: Longmans, Green and Co. 1894.—This volume is one which opens by reminding us of our own shortcomings in comparison with the life-history of the series of great men whose "footprints on the sands of time" are sketched by Dr. W. S. Church, on stepping into the breach caused by the decease of the president, Sir Andrew Clark. The Society lost during the year no less than three Honorary Fellows and eleven Fellows, in addition to its president. The obituary notice gives interesting details of the lives of Marcus Beck, Jean Martin Charcot, William Morse Graily Hewitt, Sir Andrew Clark, John Tyndall, Professor Theodor Billroth, and several other well-known Fellows. Many of the papers which occupy the bulk of the volume are of considerable interest; but to us locally that of Mr. J. Greig Smith, on the spontaneous disappearance of solid abdominal tumours, is perhaps the most noteworthy. What excellent cases would these have been for a demonstration in Paris of the miraculous cures effected at Lourdes!

*Transactions of the American Surgical Association.* Vol. XII. Philadelphia: WILLIAM J. DORNAN. 1894.—This annual volume of the American Surgical Association furnishes us with the usual interesting report of the papers read, and the discussions which have taken place during the year. The Surgical Treatment of Empyema is introduced by John Ashhurst, jun., in a paper full of valuable suggestions. The Methods of Teaching Surgery are discussed in papers by John S. Billings and John Chiene (of Edinburgh), and in debate by J. Collins Warren, W. W. Keen, Charles B. Nancrede, and others. Papers on The Surgery of the Kidney, by Louis McLane Tiffany; The Surgery of the Ureter, by Christian Fenger; The Treatment of Inoperable Malignant Tumours with the Toxines of Erysipelas and the Bacillus Prodigiosus, by William B. Coley; and half-a-dozen others make up a volume which is full of interesting reading, and fully maintains the high reputation of its predecessors.

*Index-Catalogue of the Library of the Surgeon-General's Office, United States Army.* Vol. XV.—UNIVERSIDAD–VZOROFF. Washington: GOVERNMENT PRINTING OFFICE. 1894.—We heartily congratulate the Editors of this stupendous work on another instalment of their labours. We have on former occasions noticed in these pages various volumes as they have been published, and have each time had to express our admiration for the manner in which the work has been done, and the completeness and clearness of the references. A review, as is generally understood, is impossible of such a book as this—the value of it is tested when one makes use of it, and we do not remember in the numerous occasions on which we have turned to it ever having found a misleading reference. We commend to the notice of authors the admirable way in which the references are made, with a hope that they may read, mark, and learn.

*The Year-Book of Treatment for 1895.* London: CASSELL AND COMPANY, LIMITED.—This useful volume requires no introduction to the profession, but it certainly well deserves the welcome that has been accorded to it in the past. The information under the various headings is well arranged and selected, but we could wish that some of the sections were more complete and that the index was fuller.

*The Medical Annual.* Bristol: JOHN WRIGHT & Co. 1895. —This well-known and useful volume is built up on lines very similar to previous years. It is a worthy successor of those which have preceded it. The names of those who contribute the various articles are a sufficient guarantee that the literature of our own and other countries, periodical and otherwise, has been freely laid under contribution. The dictionary of new remedies and the articles on electro-therapeutics and on anti-microbic treatment, are specially interesting and useful parts of the work. We should like to see the 150 pages of advertisements arranged in absolute isolation from the volume itself: they have their field of utility, but they do not assist in the finding of the title-page, the preface, and the table of contents.

*The Practitioner.* Edited by MALCOLM MORRIS. London: CASSELL AND COMPANY, LIMITED. [January—June,] 1895.—We have received the first volume of the new series of this old friend, and have to congratulate the present Editor on the revived vigour of his new charge. The various modifications he has introduced are certainly likely to prove attractive: the summary of the month, and the Medico-Literary Causerie have been quite delightful reading, and we are gratified to find that their merit has been duly attested by the signs of increasing favour which the efforts of the Editor are month by month receiving; we wish him continued and increasing success in a work which must demand much time and talent. By some unaccountable oversight, the number of the volume is not given.

*Visiting List.* Third Edition. Bristol: JOHN WRIGHT & Co. 1896.—This list is now published in two forms—one has all dates, the other has the pages for visits in what is called "perpetual" form. Various minor improvements have been made, and we have no doubt that this visiting list will become increasingly popular. It deserves to hold a leading place amongst all the lists which have been devised. After an experience of three years, we can give ample testimony to its great convenience and utility.

